# The Study of the Internal Secretions

**Published:** 1916-05

**Authors:** 


**The Study of the Internal Secretions.**

Editor Buffalo Medical Journal:—

The increasing appreciation of the importance of the glands of internal secretion and their influence upon the etiology as well as the treatment of many disorders, has made the subject of unusual interest to many physicians. It has been suggested recently by several American physicians that it might be well to form an **Association for the Study of the Internal Secretions**; and it is desired to know whether there is sufficient interest in this matter to warrant its further consideration.

No effort has yet been made to form such an association; but any physicians who are interested and would welcome the establishment of a community of interest embracing some or all of the points just mentioned, as well as others which cannot be enumerated for lack of space, are requested to send their names and addresses on a postal card to the undersigned at 715-19 Baker Detwiler Bldg., Los Angeles, Cal.

HENRY R. HARROWER, M. D.

---

Dr. A. L. Benedict,

Dear Sir:—

A sphygmomanometer was left in our store recently, and we have been advised by a doctor to notify you so that you could make the fact known through the Buffalo Medical Journal.

Yours truly,

OTTO ULBRICH CO.

